# CalVSP: a program for analyzing the molecular surface areas, volumes, and polar surface areas

**DOI:** 10.1186/s13321-025-01120-2

**Published:** 2025-12-29

**Authors:** Yuzhu Li, Daiju Yang, Qingyi Shi, Weidong Zhang, Qingyan Sun

**Affiliations:** 1 School of Pharmaceutical Sciences, Shanghai Jiao Tong University, Shanghai, 200240 China; 2 National Key Laboratory of Lead Druggability Research, Shanghai Institute of Pharmaceutical Industry, China State Institute of Pharmaceutical Industry, Shanghai, 201203 China; 3 School of Pharmacy, Second Military Medical University, Shanghai, 200433 China

**Keywords:** Computer programs, Molecular surface area, Molecular volume, PSA

## Abstract

The molecular volume, surface area, and polar molecular surface area are important descriptors for characterizing and predicting the molecular properties of lead compounds. Existing computational tools for calculating the above parameters often have complex workflows and are not well-suited for high-throughput conditions. CalVSP is an open-source software for computing molecular volume, molecular surface area, and polar surface area. The software implements a grid-based algorithm that dynamically optimizes grid spacing via quantum chemical reference data to ensure precise parameter calculations. CalVSP was tested on 9489 3D molecular structures, and the results revealed a mean absolute percentage error of 1.25% (95% CI: 1.23–1.27%) for the molecular volume and 1.33% (95% CI: 1.31–1.35%) for the molecular surface area compared with the quantum chemical data. For the molecular polar surface area calculations, the mean absolute percentage error was 4.59% (95% CI: 4.16–5.04%) across the 388 tested molecular structures. The CalVSP written in the C programming language offers a lightweight and easy tool. It can be integrated with other molecular property prediction tools to increase computational accuracy and for large-scale molecular calculations.

**Graphical Abstract:**

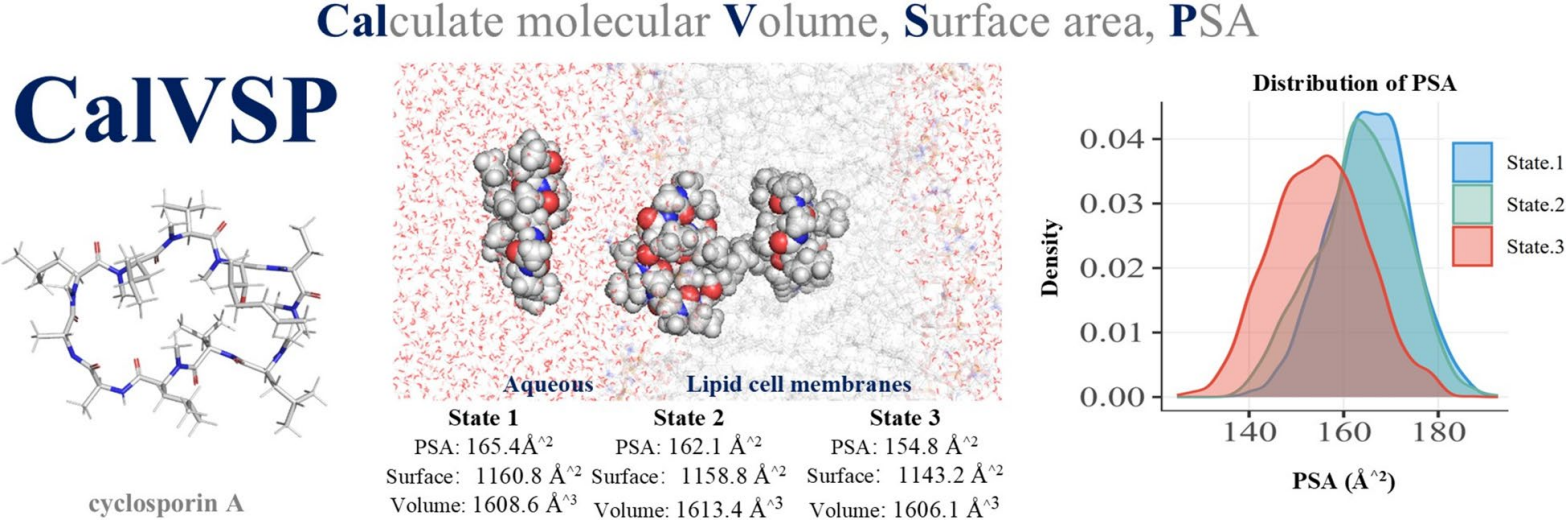

**Supplementary Information:**

The online version contains supplementary material available at 10.1186/s13321-025-01120-2.

## Scientific contribution

We provide a user-friendly, open-source command-line tool and library for calculating the molecular volume, surface area, and polar surface area. Compared with other software programs based on quantum chemical calculations, CalVSP results in fewer calculation errors and faster processing speeds. Additionally, CalVSP can be integrated as a library function and combined with other programs for advanced data mining and calculations.

**Supplementary Information:**

The online version contains supplementary material available at 10.1186/s13321-025-01120-2.

## Introduction

Molecular descriptors play an important role in predicting the physicochemical properties of molecules, drug property prediction, and noncovalent interactions between molecules. For example, the calculated molecular volume can be used to accurately describe the molecular size and space occupancy. Molecular volume, as an important alternative parameter for molecular shape analysis, can be used to calculate LogP, chromatographic capacity factor[1, 2]. The molecular surface is an approximation of the actual molecular boundary and is an important geometric parameter for describing molecules. The strength of intermolecular dispersion energy is related to the size of the contact surface area between the ligand and receptor, which also dictates their steric complementarity. This relationship between contact area and dispersion energy has been demonstrated experimentally[3]. It enables the quantitative assessment of polar surface area (PSA) exposure ratios to calculate ClogP and brain permeability in drug design[4]. The molecular volume and surface area are also used to predict the density, viscosity, heat capacity, and other physicochemical properties of ionic liquids[5]. PSA refers to the surface area occupied by oxygen or nitrogen atoms or hydrogen atoms attached to oxygen atoms (OH) or nitrogen atoms (NH) in a molecule (some scholars argue that sulfur atoms or hydrogen atoms attached to sulfur atoms (SH) should also be included). TPSA (topological PSA) is a methodology to calculate molecular PSA as a sum of polar fragment contributions[6]. PSA and TPSA play a critical role in the early stage of drug development, particularly in predicting drug permeability. The advantage of TPSA is straightforward calculation and does not require computationally demanding steps. Both PSA and the solvent-accessible polar surface area (SAPSA) are more effective measures of polarity than hydrogen bond donor/acceptor counts or the TPSA[7–9]. Studies on antibacterial or antiviral drugs beyond Lipinski’s rule of 5 (bRo5) have demonstrated a strong correlation between PSA and cellular permeability/solubility[7]. The permeability across monolayers of the efflux-inhibited colorectal adenocarcinoma cell line Caco-2 is correlated with the minimum SAPSA for each drug (r^2^ = 0.90) but has a weaker correlation with the drug TPSA (r^2^ = 0.36)[7, 8]. Some drugs undergo conformational changes in environments with different polarities (e.g., transitioning from aqueous phases to cellular phospholipid bilayers), especially cyclic peptides, macrocyclic drugs, and proteolysis-targeting chimeras (PROTACs), and approximately 50% of drug-like bioactive compounds exhibit this ability, termed “molecular chameleon” properties [10, 11]. In addition to changes in molecular conformation, molecules also change their protonated state with different environments. For example, protonated local anesthetics penetrate the cell membrane in a deprotonated form[12, 13]. Molecules with molecular chameleon properties can change PSA in environments with different polarities, improving the absorption characteristics and efficacy of drugs [7, 11]. PSA has become a vital descriptor for designing and researching bRo5 drugs in early drug development. Calculation of the molecular volume, surface area, and PSA is critically important for guiding drug design, physicochemical property prediction, and material performance optimization.

Molecular volume, which is an unobservable measure referring to the space enclosed by molecular surfaces, inherently has varying definitions across calculation methods, leading to discrepancies in computed volumes owing to differing surface models. The commonly used molecular volume is the van der Waals volume. The van der Waals volumes and surface areas of molecules can be calculated by summing the atomic sphere volumes and surfaces or through Monte Carlo simulation algorithms by predefined atomic van der Waals radii[14]. The van der Waals volumes of molecules change due to the electronic effects of electron transfer and electron cloud distortion during chemical bond formation. Calculating the molecular van der Waals volume by summing the atomic sphere volumes may underestimate the impact of electronic effects on these values. Nevertheless, this computationally efficient method is particularly suitable for large molecules such as polymers, proteins, and nucleotides, offering significant advantages in the calculation of volume and surface area. Different research methodologies lead to discrepancies in atomic van der Waals radius data[15–17]. Consequently, the calculated molecular van der Waals volume and surface area variations are based on different radii.

Different studies have different definitions for isosurface, Bader proposed an isosurface with an electron density of 0.001 a.u. for van der Waals surfaces in the gas phase and 0.002 a.u. for the solid phase[18–20]. This molecular surface definition, which accounts for more than 98% of the electron density distribution and addresses the limitations of atomic sphere superposition methods (which neglect electronic effects), has been widely adopted in computational chemistry. Calculating van der Waals volumes through Bader's definition involves two steps: (1) performing density functional theory (DFT) calculations on the molecule's 3D structure to obtain its wavefunction and (2) applying Monte Carlo integration or marching tetrahedron algorithms to calculate the volume or surface[21].

However, generating wavefunction files via quantum chemistry (QC) calculations is computationally demanding and requires substantial resources and specialized software. As the number of atoms in a molecule increases, the required computation time and memory demand increase substantially, rendering this method inefficient for large-scale molecular systems or high-throughput computational studies.

Many software programs are available for the calculation of molecular volume, surface area, and PSA. For example, the excellent open-source MoloVol can calculate molecular cavities, volumes, and surfaces, demonstrating high efficiency for large molecules such as protein structures[22]. Additionally, Zeo +  + and PoreBlazer are widely used for molecular volume/surface area calculations[23, 24]. As visualization software, PyMOL provides functionalities for van der Waals or solvent-accessible surface area computations[25]. PLATON can support molecular volume calculations[26]. Polarsa2 calculates the PSA by processing van der Waals surface data generated via MOLVOL[27]; however, its workflow remains cumbersome and currently only supports PSA calculations. The QC analysis software Multiwfn can analyze QC data to extract molecular volume, surface area, and PSA data[28]. However, the above software has drawbacks of computational inefficiency and procedure complexity or the inability to calculate these three parameters with the same software when calculating the molecular volume, surface area, and PSA.

To overcome the above shortcomings, we introduce CAlVSP, a free program for calculating molecular volume, surface area, and PSA. The tool is primarily based on a grid algorithm to approximate the Bader van der Waals volume or surface area and calculate the van der Waals volume, surface area, and PSA of molecules. CalVSP has significant advantages, including high accuracy, as well as improved computational efficiency relative to that of QC workflows. Thus, CalVSP is particularly suitable for calculating the molecular volume, surface area, and PSA of high-throughput or high-throughput molecules with many atoms. The surface area, volume, and PSA of certain molecules may be influenced by their molecular configurations and vary among various conformational states, thus requiring large-scale conformational sampling and analysis. Existing software in such scenarios often suffers from cumbersome or time-consuming workflows, whereas CalVSP efficiently handles these computations. For large-scale conformational sampling studies, CalVSP supports direct processing of molecular conformational trajectory files in the xyz format, enabling rapid batch calculations.

CalVSP is an open-source application written in C. The entire source code is available on a repository hosted on GitHub https://github.com/CalVSP/CalVSP.git and is free to use and modify under the MIT license. The application is operated via a command‒line interface. CalVSP has been tested on Windows 11 and Ubuntu 20.04 LTS. Current and past releases are available as precompiled binaries or as source code via https://github.com/CalVSP/CalVSP.git. Future releases will be made available through the same web page.

## Implementation

**Fig. 1:**
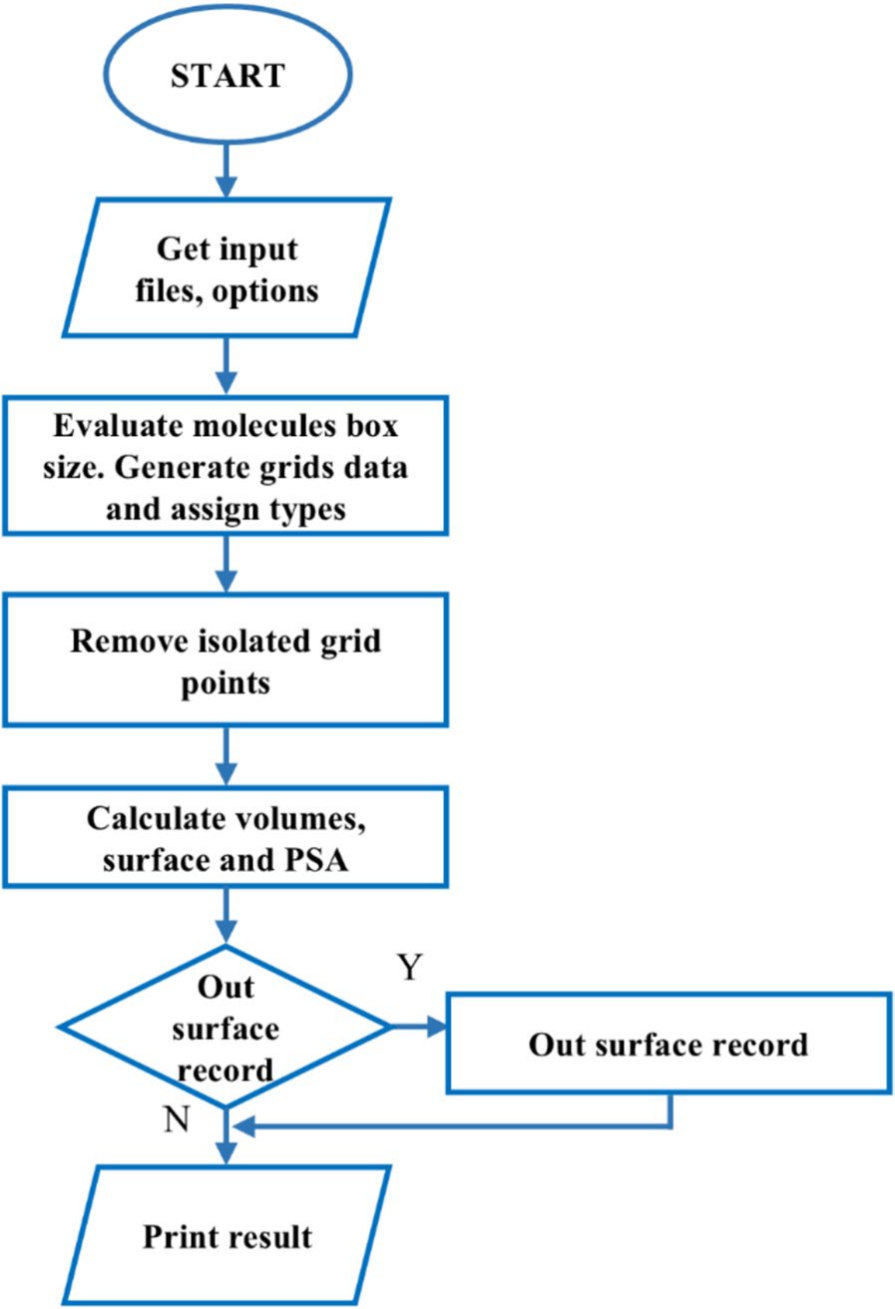
Flow chart presenting the CalVSP calculation algorithm

The overall calculation process of CalVSP is illustrated in Fig. 1. Users may submit 3D molecular structure files in mol2, pdb, sdf, or xyz formats to perform calculations of molecular van der Waals or solvent-accessible surface area, PSA, and volume with CalVSP. CalVSP identifies the file type on the basis of the input file's extension and subsequently extracts atomic information along with corresponding Cartesian coordinates, applying format-specific parsing rules. CalVSP extracts atomic information from different molecular file formats based on chemical record formats, and its core process is as follows: For MOL2 format: The program reads the data between @ < TRIPOS > ATOM and @ < TRIPOS > BOND field identifiers for calculation. For PDB format: The program extracts atomic information from all standard record lines starting with the keywords "ATOM" or "HETAMM". For SDF format: The program first obtains the total number of atoms from the third line of the file, and then uses this value as the upper limit to read the atomic coordinate data of the corresponding line number. For XYZ format: The program parses the total number of atoms declared on the second line of the file and reads the atomic coordinates corresponding to the subsequent lines. For trajectory files containing multiple conformations, CalVSP will parse them frame by frame and calculate for each frame. This method only applies to parsing and extracting data explicitly declared in files. The entire process does not involve the determination of chemical bonding relationships within molecules or the number of molecules in the document.

Once the data are acquired, the system calculates the van der Waals or solvent-accessible volume, surface area, and PSA of the molecule.

## Calculation details

### Selection of functional/basis set and grid resolution

The selection of different functional/basis set combinations can significantly affect the computation time, memory requirements, and accuracy of the results. For testing purposes, we conducted calculations on 23 molecular compounds via 19 distinct functional/basis set combinations (Table 1) and generated corresponding wavefunction files. Using Multiwfn software with an electron density isosurface threshold of 0.001 a.u. and a spacing of grid points of 0.15 Bohr, we calculated 3D molecule volumes [28].

**Fig. 2:**
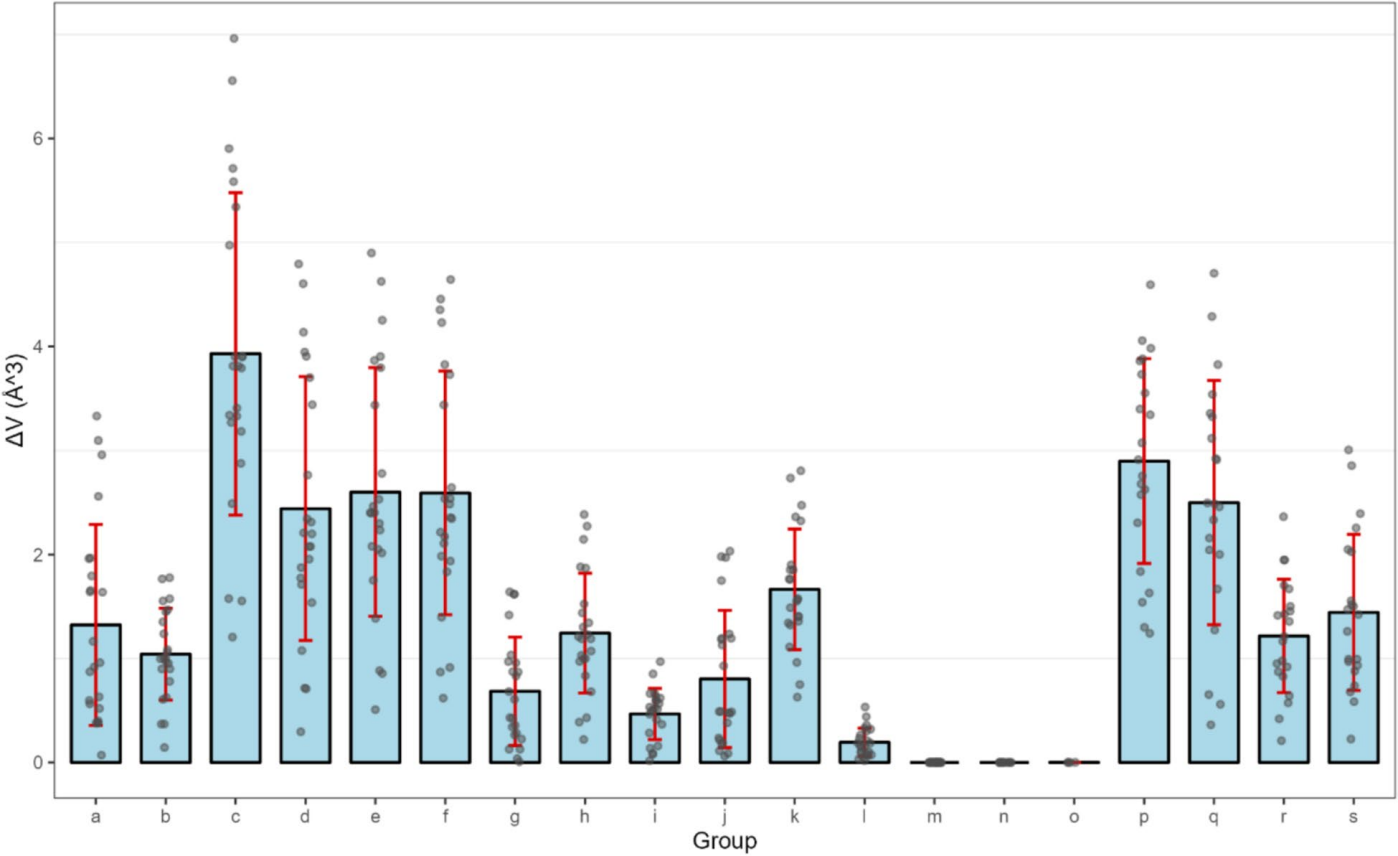
Absolute errors in molecular volume compared with the m-functional/basis set

To evaluate the accuracy of the volumes obtained from different functional/basis set combinations calculated, a theoretical benchmark was needed. We employed the highly accurate DLPNO-CCSD(T)/cc-pVTZ, which provides results close to the CCSD(T) complete basis set as the reference[29, 30]. The absolute errors in the molecular volume calculations were then determined by comparing the values from the 18 functional/basis set combinations against this DLPNO-CCSD(T) benchmark. As shown in Fig. 2, the b group r2SCAN-3c functional demonstrates the lowest absolute error in the results of the molecular volume and achieves faster computational speeds than other functional/basis set combinations. Consequently, the r2SCAN-3c-derived results were adopted as reference standards to optimize the grid resolution parameters to minimize errors.

**Table 1:** Selected functional basis sets

Group	Functional basis sets
a	B97-3c [31–34]
b	r2SCAN-3c [35–37]
c	BLYP D3 def2-TZVP [31, 32, 38, 39]
d	B3LYP D3 def2-TZVP(-f) [31, 32, 38–40]
e	B3LYP D3 def2-TZVP [31, 32, 38, 39]
f	wB97M-V def2-TZVP [38, 39, 41–43]
g	PWPB95 D3 def2-TZVPP [31, 32, 38, 39, 44]
h	wB97X-2 D3 def2-TZVPP [31–33, 38, 39, 45]
i	D4 def2-TZVPP [37–39]
j	PWPB95 D3 def2-QZVPP [31, 32, 38, 39]
k	wB97X-2 D3 def2-QZVPP [31–33, 38, 39]
l	D4 def2-QZVPP [37–39]
m	DLPNO-CCSD(T) normalPNO [46]
n	DLPNO-CCSD(T) tightPNO [46]
o	CCSD(T) [46]
p	B3LYP/6-31G[47–51]
q	B3LYP/6-31G**[47–52]
r	B3LYP/6-311G[47, 48, 51, 53]
s	B3LYP/6-311G** [47, 48, 51, 53]

We selected 3D molecules with different sizes of *V*_rec_ (the volume of the rectangular bounding box) and compared the molecular volumes calculated with different spacings of grid points against QC-derived benchmarks to identify the spacing of grid points of volume error minimized for different sizes of *V*_rec_.

The *V*_rec_ is defined by Eq. (1) for molecular volume calculations, via enumerating the Cartesian coordinate values of all the atoms in a molecule and determining the maximum and minimum values along the *X*-, *Y*-, and *Z*-axes. Here, *x*_max_, *x*_min_, *y*_max_, *y*_min_, *z*_max_, and *z*_min_ represent the maximum and minimum coordinate values of all the atoms A_*i*_(*x*_*i*_, *y*_*i*_, *z*_*i*_) of the molecule.

1 $$ V_{{{\text{rec}}}} = \prod\limits_{{k \in \{ x,y,z\} }} {(k_{{{\text{max}}}} - k_{{{\text{min}}}} )} $$

The relationship between *V*_rec_ with minimal error and the spacing of grid points was established, with specific values tabulated in support information **Table S1**.

### Generating grid points and calculating data values

Then, molecules are encapsulated in rectangular boxes of suitable dimensions, and three-dimensional grid point data **P**(*x,y,z*) are generated in Cartesian coordinates using the predefined grid resolution. Each grid point **P**_*j*_ (*x*_*j*_*, y*_*j*_*, z*_*j*_) is scanned across 26 directions (6 orthogonal, 12 face-diagonal, and 8 body-diagonal) to compute the minimum Euclidean distance *R*_dis_ to the nearest atom **A**_*i*_ (*x*_*i*_*, y*_*i*_*, z*_*i*_). *R*_dis_ is compared with the van der Waals radius *R*_vdw_ of the nearest atom according to Eq. (2)[54]:

2 $$ R_{{{\text{dis}}}} = \min_{{{\mathbf{A}}_{i} }} \sqrt {(x_{{\mathbf{P}}} - x_{i} )^{2} + (y_{{\mathbf{P}}} - y_{i} )^{2} + (z_{{\mathbf{P}}} - z_{i} )^{2} } $$

The grid points are classified as follows:

- Internal if *R*_dis_ < *R*_vdw_,
- Surface if *R*_dis_ = *R*_vdw_ ​,
- External if *R*_dis_ > *R*_vdw_.

This classification is mathematically defined as Eq. (3):

3 $$ {\text{Mark}}({\mathbf{P}}_{i} ) = {\text{sign}}(R_{{{\text{vdw}}}} - R_{{{\text{dis}}}} ) = \left\{ {\begin{array}{*{20}c} {\begin{array}{*{20}c} 1 \\ 0 \\ { - 1} \\ \end{array} } & {\begin{array}{*{20}c} {{\text{if }}R_{{{\text{dis}}}} < R_{{{\text{vdw}}}} } \\ {{\text{if }}R_{{{\text{dis}}}} = R_{{{\text{vdw}}}} } \\ {{\text{if }}R_{{{\text{dis}}}} > R_{{{\text{vdw}}}} } \\ \end{array} } \\ \end{array} } \right. $$

Isolated misassigned grid points (e.g., P_*j*_) at the center of 3 × 3 × 3 cubic lattices are scanned 26 neighboring grid points (including face-, edge-, and corner-sharing grid points in 3D space). When any grid point P_*j*_ exhibits the condition where in one or more of its 13 neighboring directions, the two opposing grid points P_*j1*_ and P_*j2*_ along such a direction possess the same attributes that differ from P_*j*_’s own, P_*j*_ undergoes reclassification so that its attributes align with those of its neighbors (P_*j1*_ and P_*j2*_). This correction process is similar to that of the flood-fill algorithm in that all grid points are accurately classified, and it is iterated until there are no inconsistencies.

### Molecular volume and surface area calculations

The volume weight factor is defined by summing the contributions of the internal and surface grid points via Eq. (4); then, the molecular volume *V* can be calculated via multiplication with the unit grid volume via Eq. (5):

4 $$ {\mathbf{G}}_{V} = \mathop \sum \limits_{j = 1}^{N} \left\{ {\begin{array}{*{20}c} 1 & {{\text{if Mark}}({\mathbf{P}}_{j} ) \ne - 1} \\ 0 & {{\text{otherwise}}{. }} \\ \end{array} } \right. $$

5 $$ V = {\mathbf{G}}_{V} \times {\text{grid}}\_{\text{size}}^{{3}} $$

Similarly, the surface area weight factor is defined by Eq. (6), and the molecular surface area *S* can be empirically corrected via QC benchmarks according to Eq. (7):

6 $$ {\mathbf{G}}_{S} = \mathop \sum \limits_{j = 1}^{N} \left\{ {\begin{array}{*{20}c} 1 & {{\text{if Mark}}({\mathbf{P}}_{j} ) = 0} \\ 0 & {{\text{otherwise}}{. }} \\ \end{array} } \right. $$

7 $$ S = {\mathbf{G}}_{S} \times {\text{grid}}\_{\text{size}}^{{2}} \times 1.261 + 13.506 $$

### PSA calculation

PSA is defined as the surface area contributed by polar atoms (N, O, NH, OH). For each surface grid point P_*suf*_, the nearest atom is identified. If it belongs to the polar group, P_*suf*_ is labeled P_PSA_ according to Eq. (8):

8 $$ {\text{Mark}}({\mathbf{P}}_{{{\text{PSA}},i}} ) = \left\{ {\begin{array}{*{20}c} 1 & {R_{{{\text{dis}}}} = R_{{{\text{vdw}}}} \wedge {\mathbf{A}}_{i} \in \{ {\mathbf{N}},{\mathbf{O}},{\mathbf{NH}},{\mathbf{OH}}\} } \\ 0 & {{\text{otherwise}}{. }} \\ \end{array} } \right. $$

The PSA *S*_PSA_ is then calculated according to Eqs. (9) and (10):

9 $$ {\mathbf{G}}_{{\mathbf{P}}} { = }\mathop \sum \limits_{j = 1}^{N} \left\{ {\begin{array}{*{20}c} 1 & {{\text{if Mark}}({\mathbf{P}}_{{{\text{PSA,}}j}} ) = 1} \\ 0 & {{\text{otherwise}}{. }} \\ \end{array} } \right. $$

10 $$ S_{{_{{{\text{PSA}}}} }} = \frac{{{\mathbf{G}}_{{\mathbf{P}}} }}{{{\mathbf{G}}_{{\mathbf{S}}} }} \times S $$

### Output molecular surface information

The CalVSP program outputs results according to computational requirements and exports surface grid data in the xyz format for downstream applications.

## Results and discussion

### Determination of Optimal Grid Spacing

Our initial investigation on a parameterization set of 57 molecules revealed that the grid spacing which minimizes systematic error for an individual molecule is influenced by its specific physical characteristics (e.g., shape, compactness), leading to a distribution of molecule-specific optimal values (see Table S1, Supplementary Information). The arithmetic mean of these 57 optimal values is 0.43 Å, and they were found to cluster around this common value. This suggested that a single, universal grid spacing of 0.43 Å might serve as a robust approximation for the molecule-specific optima.

To rigorously test this hypothesis and assess the generalizability of both approaches, we performed a large-scale validation on an independent set of 9,489 molecules. We evaluated the performance of the universal spacing (0.43 Å) against our size-dependent parameterization. The results are summarized in Table 2.

**Table 2:** Comprehensive performance comparison on independent test set (n = 9489)

Metric	Size-dependent (Table S1)	Universal 0.43 Å spacing
Volume RMSE (Å^3^)	6.44 ± 0.05	6.06 ± 0.05
Surface Area RMSE (Å^2^)	6.22 ± 0.05	6.31 ± 0.05

The uncertainty estimates ( ±) for the test set were obtained via bootstrapping

As shown in Table 2, the validation reveals:

The universal spacing (0.43 Å) provides superior accuracy for molecular volume calculation on the independent test set. Conversely, the size-dependent parameterization delivers the highest accuracy for molecular surface area calculation.

Given that the precise computation of molecular surface area is the primary requirement of our analysis and is central to the conclusions of this work, we have elected to retain and recommend the size-dependent scheme. This approach is justified by its optimal performance for our key metric of interest. The universal spacing of 0.43 Å remains a simplified alternative for applications where volumetric accuracy is the priority.

### Comparison of the CalVSP and QC methods for molecular surface area and volume calculations

Since existing molecular volume and surface area calculation tools (nonquantum calculation methods) fail to consider changes in molecular volume and surface area caused by electron cloud redistribution during interatomic bonding, we compared CalVSP computational accuracy against reference values obtained from quantum chemical calculations. Our calculations show that these changes are significant: for example, in molecules like CO₂, the volume decreases by approximately 35.8% compared to the sum of atomic volumes, while the surface area decreases by 45.9% (see Table S3 for details).

Test dataset preparation: To ensure a random selection, 11,000 Compound ID (CID) numbers were randomly sampled from the PubChem database. A total of 10,000 corresponding 3D compound structures were then downloaded in SDF format [55]. All molecular structures that successfully completed the quantum chemical calculation workflow (n = 9489) were programmatically validated to confirm non-zero Z-coordinates for all atoms, ensuring the use of authentic 3D geometries in subsequent analysis. Wavefunction files were calculated via the ORCA 5.0.3 program (r2SCAN-3c functional, no structure optimization) and processed at Multiwfn 3.8 (isosurface threshold: 0.001, 0.002 a.u*.*, grid point spacing: 0.15 or isosurface threshold: 0.0016 a.u., grid point spacing: 0.15) to obtain QC-derived molecular volumes and surface areas[20, 56–60].

In response to reviewer comments, we have enhanced the CalVSP software to support multiple isosurface thresholds (0.001, 0.0016, and 0.002 a.u.), thereby improving its versatility. To validate our methodology against the benchmark established by Amin Alibakhshi[60] (which employs DSD-PBEP86 0.0016 a.u.), we confirmed the agreement between r2SCAN-3c and DSD-PBEP86 functionals under identical conditions. Detailed comparisons are provided in Supplementary Material (Fig. S1).

After filtering out invalid or incomplete entries, the volume and surface area values of 9489 compounds were obtained through the above calculation process. The corresponding CalVSP calculations are performed on the same dataset. A comparative analysis was performed between the CalVSP and QC results.

In this work, the statistical parameters mean squared error (MSE, Eq. (11)), root mean squared error (RMSE, Eq. (12)), mean absolute error (MAE, Eq. (13)), absolute percentage error (APE, Eqs. (14,15)), and mean absolute percentage error (MAPE, Eq. (16)) were employed to evaluate the calculation results.

11 $$ {\text{MSE}} = \frac{1}{n}\sum\limits_{k = 1}^{n} {(CalVSP_{k} - QM_{k} )^{2} } $$

12 $$ {\text{RMSE}} = \sqrt {{\text{MSE}}} $$

13 $$ {\text{MAE}} = \frac{1}{n}\sum\limits_{k = 1}^{n} {\left\| {CalVSP_{k} - QM_{k} } \right\|} $$

14 $$ {\text{Reltative Error(}}k{) = }\frac{{CalVSP_{k} - QM_{k} }}{{QM_{k} }} $$

15 $$ {\text{APE}}_{k} = \left\| {{\text{Relative Error}}(k)} \right\| \times 100\% $$

16 $$ {\text{MAPE}} = \frac{1}{n}\sum\limits_{k = 1}^{n} {\left\| {{\text{APE}}_{k} } \right\|} $$

**Fig. 3:**
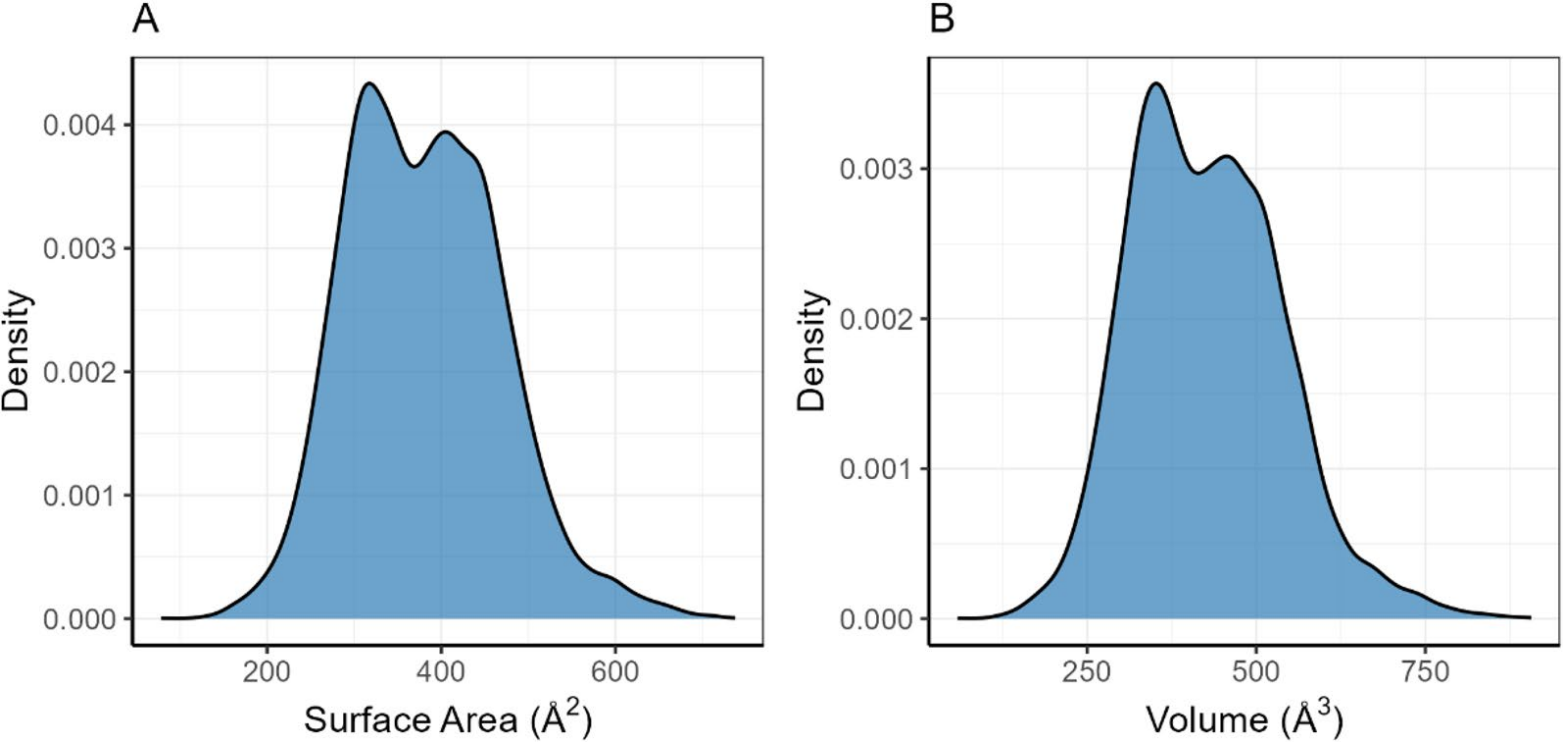
**A** QC-calculated vdW surfaces frequency distribution; **B** QC-calculated vdW Volumes frequency distribution;

The frequency distributions of QC-calculated van der Waals surface areas and volumes are presented in Fig. 3. Both the surface areas (Fig. 3A) and volumes (Fig. 3B) distributions exhibit distinct bimodal characteristics, reflecting the structural diversity of the molecular dataset. The surface areas distribution spans from 78.33 to 737.27 Å^2^ with a mean of 380.19 Å^2^, while the volumes distribution ranges from 58.30 to 906.83 Å^3^ with a mean of 426.62 Å^3^.

**Fig. 4:**
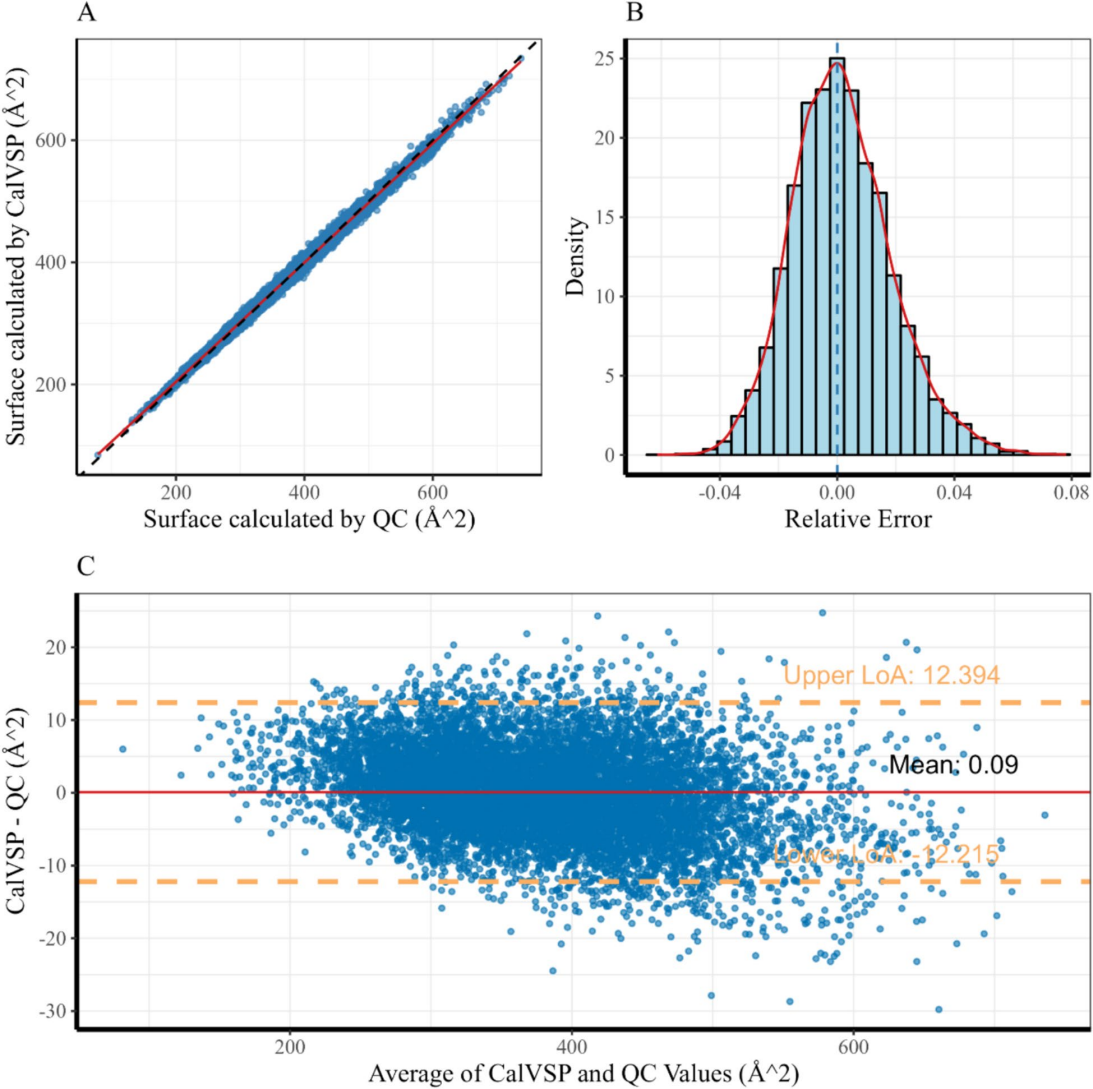
Computational validation of van der Waals surface metrics: **A** correlation scatter plot between QC-derived and CalVSP-calculated vdW surfaces; **B** relative error frequency distribution of surface area computations; **C** Bland‒Altman analysis of methodological agreement;

For surface area computations, regression analysis revealed a strong correlation between the QC- and CalVSP-derived surface areas (R^2^ = 0.99503, Pearson's r = 0.9976; N = 9489), as shown in Table 3. The relative error distribution (Fig. 4B) revealed 95% values within [− 2.94%, + 3.91%] (full range: − 6.14% to + 7.81%), with relative errors tightly clustered around neutrality (median = 0.046%) and a standard deviation (SD) of 1.71%. As shown in Fig. 4C and Table 3, the Bland–Altman analysis demonstrated minimal systematic bias (mean residual = 0.09 Å^2^, MAE = 4.91 Å^2^), and 94.8% of the differences fell within the 95% limits of agreement (LoA: ± 1.96σ, σ = 6.28 Å^2^).

**Table 3:** Comparison between CalVSP and QC calculation methods in calculating molecular surface area and volume

Metric	Surface	Volume
MSE	38.7 ± 0.6 (Å^2^)^2^	41.5 ± 0.6 (Å^3^)^2^
RMSE	6.22 ± 0.05 Å^2^	6.44 ± 0.05 Å^3^
MAE	4.91 ± 0.04 Å^2^	5.18 ± 0.04 Å^3^
MAPE	1.33 ± 0.01%	1.251 ± 0.009%
Pearson’s r	0.99767 ± 0.00005	0.9986 ± 0.00003
*R*^2^	0.99503 ± 0.0001	0.99669 ± 0.00007

The uncertainty estimates ( ±) were obtained via bootstrapping

**Fig. 5:**
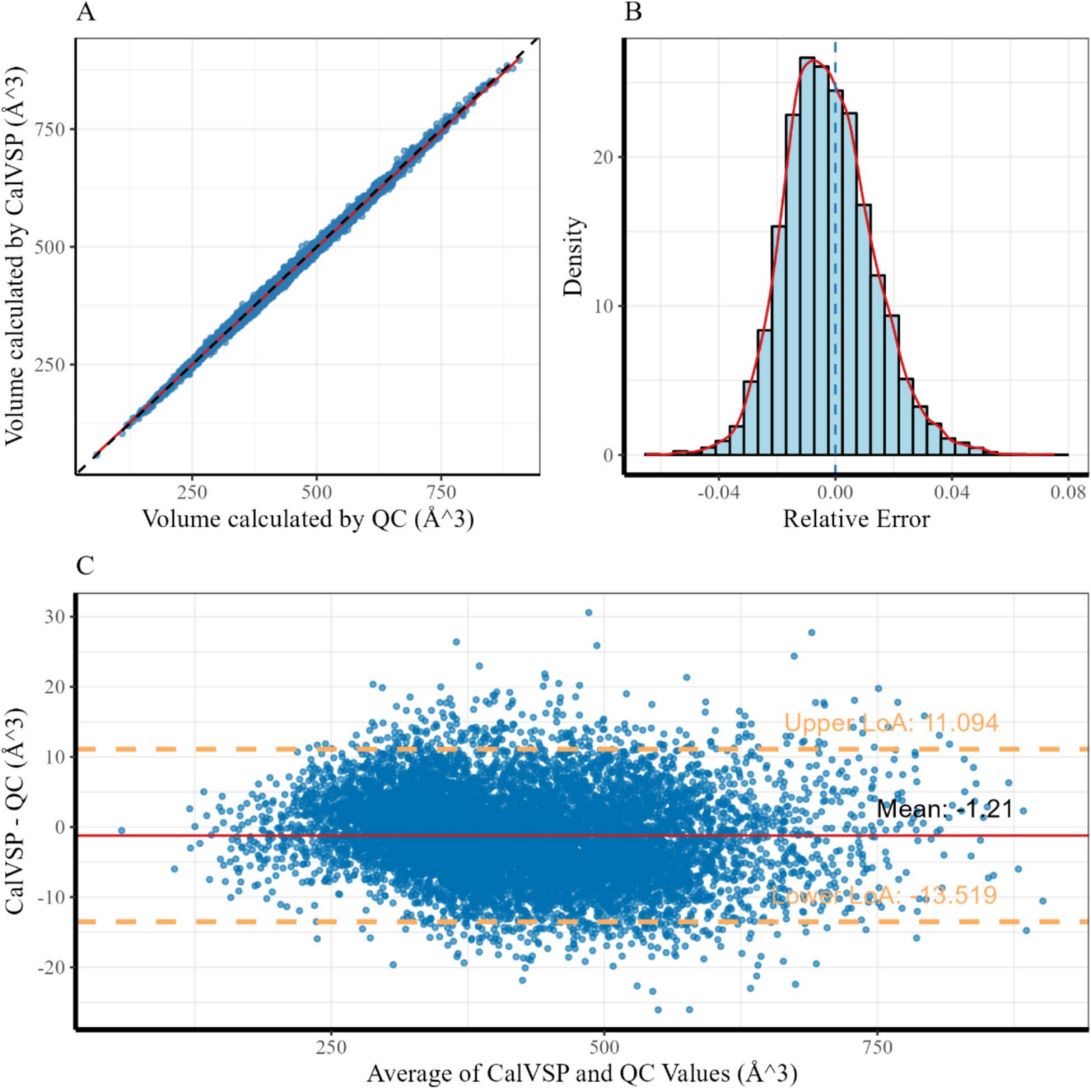
Computational validation of van der Waals volume metrics: **A** Scatter plot comparing molecular van der Waals volumes derived from QC calculations and CalVSP; **B** Relative error distribution of CalVSP-calculated van der Waals volumes relative to that of QC; **C** Bland‒Altman plot assessing the agreement between the van der Waals volumes calculated via QC and CalVSP;

Regression analysis revealed a strong correlation between the QC- and CalVSP-calculated molecular volumes, with a coefficient of determination (R^2^ = 0.99669, Pearson's r = 0.9986; N = 9489), as shown in Table 3. The relative error distribution (Fig. 5B) revealed 95% values within [− 2.95%, + 3.09%] (full range: − 6.51% to + 7.52%), with a median of − 1.21% and an SD of 1.54%, indicating slight systematic underestimation by the CalVSP method compared with the QC method while maintaining high overall reliability. As shown in Fig. 4C and Table 3, the Bland‒Altman analysis (Fig. 5C) demonstrated minimal systematic bias (mean residual = − 1.21 Å^3^, MAE = 5.18 Å^3^), and 95.0% of the differences fell within the 95% limits of agreement (LoA: ± 1.96σ, σ = 6.28 Å^3^).

To evaluate the CalVSP across different isosurface thresholds, we extended our validation to thresholds of 0.0016 and 0.002 a.u. The results (Supplementary Material: Tables S5–S6 and Figs. S2–S5) demonstrate agreement with QC calculations, with R^2^ values > 0.996 and MAPE < 1.1% for both surface area and volume at all thresholds. This confirms that CalVSP supports multiple isosurface thresholds for molecular surface area and volume computations, enhancing its versatility and practical applicability in diverse computational scenarios.

The surface of atoms and molecules is defined as an iso-density surface based on electron density data. Different studies have proposed varying threshold values, each yielding significantly different iso-density surfaces[18, 19]. For instance, Bader et al. suggested a threshold of 0.001 a.u. for gas-phase systems (e.g., methane and inert gases), where it aligns with experimentally measured equilibrium diameters [20]. In contrast, for condensed phases or van der Waals interactions (such as crystal packing or solvent-accessible surfaces), a higher threshold of 0.002 a.u. may be more appropriate[20]. Recently, Amin et al. recommended a value of 0.0016 a.u. based on thermodynamically effective (TE) surfaces, which are derived from experimental phase-change data (e.g., vaporization enthalpy and surface tension) [60]. This suggests that the optimal iso-density threshold depends on the molecular state: gas-phase systems may require a lower value (0.001 a.u.), while condensed phases need a higher value (0.002 a.u.). The 0.0016 a.u. threshold, validated against TE surfaces, is particularly suitable for liquid states, as TE surfaces incorporate liquid-phase experimental data. Consequently, to accommodate these diverse scenarios, CalVSP supports multiple iso-density thresholds (0.001, 0.0016, and 0.002 a.u.) for calculating molecular surface area and volume, enhancing its versatility across computational applications.

In summary, these quantitative assessments validate CalVSP's reliability for van der Waals surface and volume computations compared with the reference QC methods.

### Comparison of the molecular surface area, volume, and PSA across computational tools

MoloVol is an excellent tool for calculating molecular volume and surface area. PyMOL provides the molecular surface area and PSA calculations. This study evaluated the accuracy of CalVSP, MoloVol, and PyMOL by comparing their calculated molecular volumes, surface areas, and PSAs against reference values obtained from QC calculations.

Methodological Constraints:

- Molecular Volume: Due to the lack of a volumetric calculation function in PyMOL, only CalVSP and MoloVol were compared for this parameter.
- Molecular Surface Area: All three tools (CalVSP, MoloVol, PyMOL) were assessed via shared capabilities.
- PSA: MoloVol does not support direct PSA computation; therefore, comparisons were restricted to CalVSP and PyMOL.

A randomly selected subset of 390 3D molecular structures was extracted from the original 9,489 compounds with validated QC calculations. The selection protocol was based on Eqs. (17) and (18).

17 $$ n = \frac{{z^{2} \times \hat{p}(1 - \hat{p})}}{{\varepsilon^{2} }} $$

18 $$ n^{\prime} = \frac{n}{{1 + \frac{{z^{2} \times \hat{p}(1 - \hat{p})}}{{\varepsilon^{2} N}}}} $$

**Fig. 6:**
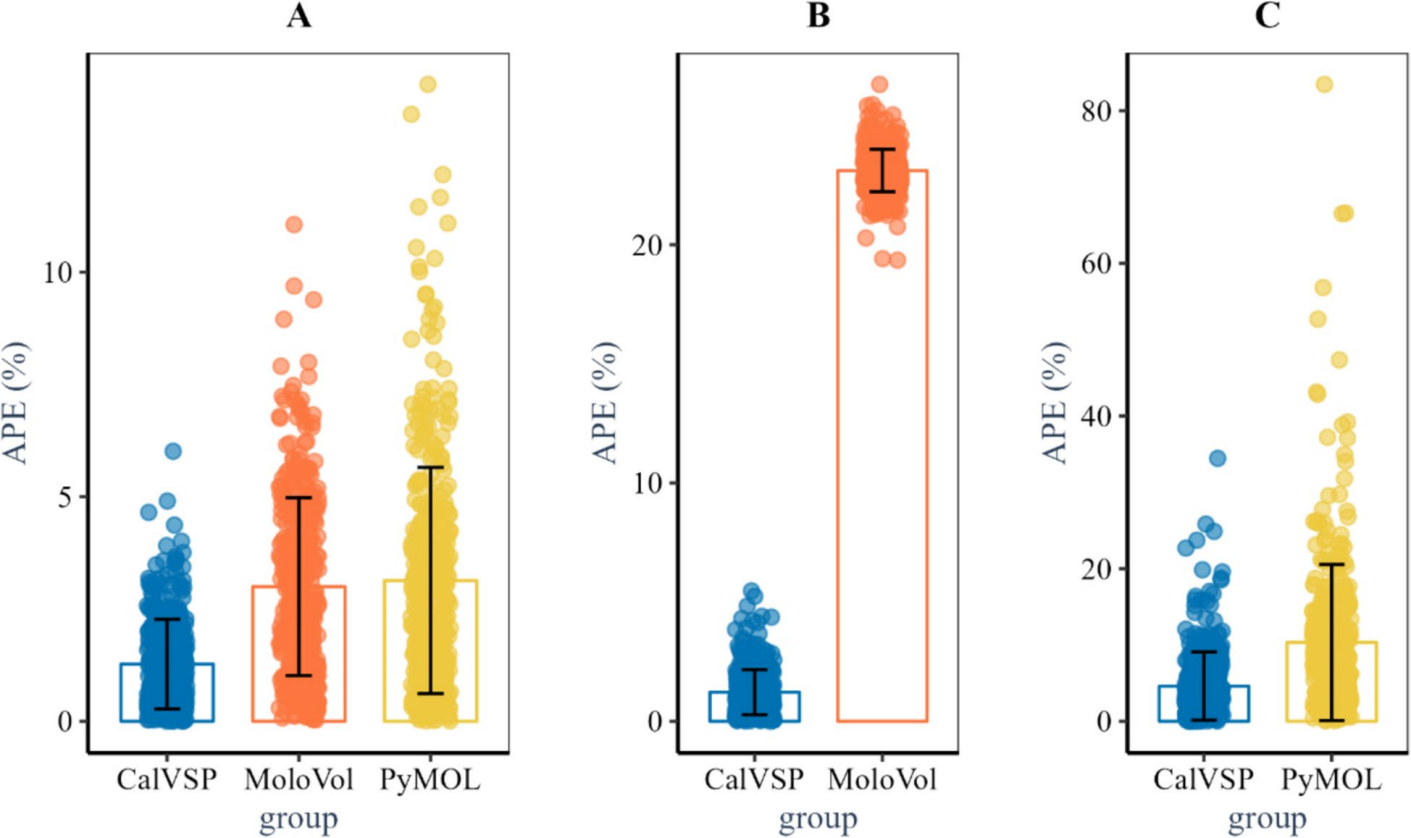
**A** Average percent error (APE) comparison of molecular surface areas calculated by CalVSP, MoloVol, and PyMOL against QC-derived reference values. **B** APE comparison of molecular volumes calculated by CalVSP and MoloVol relative to the QC benchmarks. **C** APE comparison of PSA between the CalVSP and PyMOL methods versus the QC methods

As shown in Table 4 and Fig. 6A, CalVSP-calculated molecular surface areas exhibit a MAPE of 1.27% relative to the QC reference values. Compared with MoloVol and PyMOL, CalVSP achieves better precision in surface area calculations, with both lower APEs and reduced SDs of APEs.

**Table 4:** Error analysis of CalVSP, MoloVol, and PyMOL compared with the QC reference methods

	Surface Areas			Volumes			PSAs		
	RMSE(Å^2^)	MAPE(%)	SD_APE_	RMSE(Å^3^)	MAPE(%)	SD_APE_	RMSE(Å^2^)	MAPE(%)	SD_APE_
CalVSP	6.2 ± 0.2	1.27 ± 0.05	0.016	6.4 ± 0.2	1.22 ± 0.05	0.015	3.4 ± 0.1	4.6 ± 0.2	0.062
MoloVol	12.8 ± 0.4	3.0 ± 0.1	0.032	103 ± 1	23.11 ± 0.005	0.009	–	–	–
PyMOL	16.0 ± 0.7	3.1 ± 0.1	0.039	–	–	–	7.8 ± 0.3	11.0 ± 0.8	0.196

The uncertainty estimates ( ±) were obtained via bootstrapping

For molecular volumes (Fig. 6B), CalVSP achieves a MAPE of 1.22%, whereas MoloVol shows a substantially higher MAPE of 23.11%, which is likely attributable to systematic algorithmic biases. Although the SD of the APEs for CalVSP (0.015%) is marginally greater than MoloVol’s 0.009%, this difference is negligible given CalVSP’s order-of-magnitude improvement in the mean accuracy.

In the PSA comparisons (Fig. 6C), CalVSP outperforms PyMOL, with a MAPE of 4.6%, versus PyMOL, with a MAPE of 11.0%. Compared with PyMOL, CalVSP also has a narrower error distribution (SD: 0.062%) and broader spread (SD: 0.196%).

CalVSP demonstrates statistically significant accuracy advantages over both MoloVol and PyMOL across the evaluated metrics of molecular volume, surface area, and PSA for the tested dataset.

### Testing for high-molecular-weight compounds

**Fig. 7:**
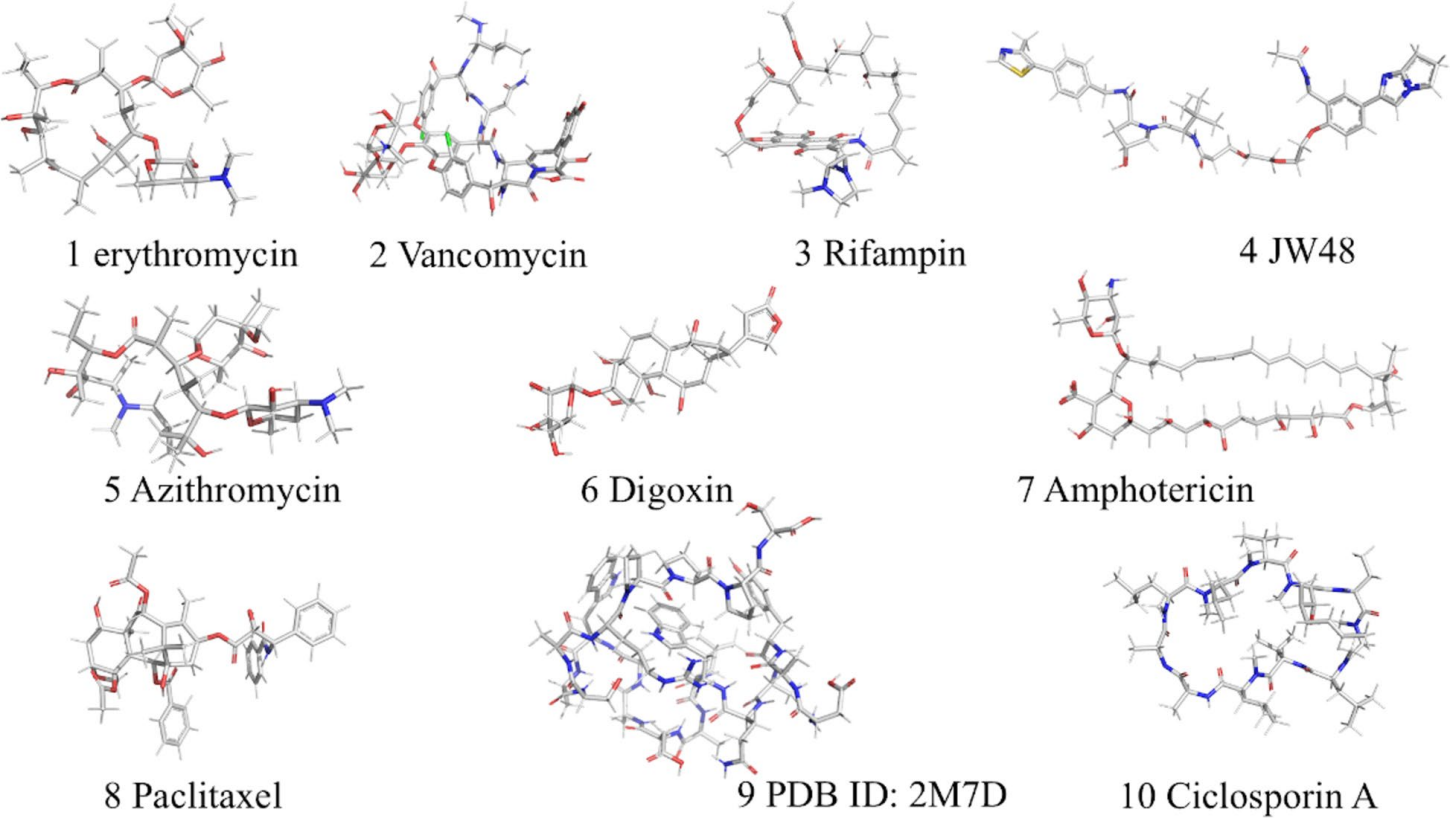
Structures of selected high-molecular-weight compounds

In prior analyses, the random selection of 3D molecular structures from the PubChem database produced a dataset with molecular atom counts primarily spanning 4–108 atoms and molecular weights within 5–780 g/mol, and the majority of compounds presented atom counts of 35–68 atoms (equivalent to molecular weights of 200–500 g/mol). To evaluate CalVSP’s applicability to larger molecules, we extended comparative calculations to high-molecular-weight compounds. However, QC methods are limited in their ability to handle large-molecule systems because of their high computational costs and memory demands. Therefore, only molecules successfully computed by QC were retained for analysis, and molecules were selected as shown in Fig. 7.

**Fig. 8:**
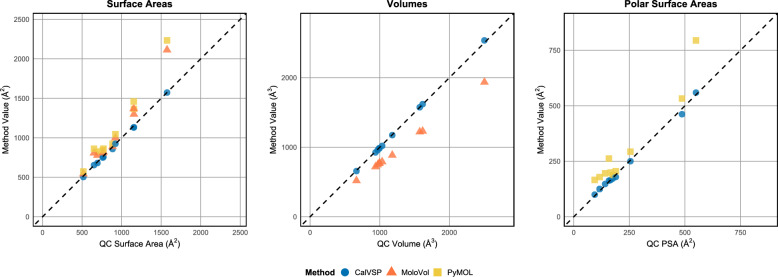
Comparison (volume, area, and PSA) between CalVSP and QC for high-molecular-weight compounds

As shown in Fig. 8, CalVSP is in closer agreement with the QC-derived results in calculating the molecular volume, surface area, and PSA for high-molecular-weight compounds, outperforming PyMOL and MoloVol. These include bRo5 drugs such as macrocyclic antibiotics, cyclic peptides, PROTACs, cardiotonic agents, antitumor compounds, and biological macromolecules such as small proteins. CalVSP, which simplifies and streamlines the molecular property calculation process by eliminating the complex input file preparation required for QC workflows, offers a more user-friendly and efficient alternative to costly QC methods.

### Calculation time test

Testing was conducted on 27 three-dimensional structured molecules with varying atom counts. The computational times required by CalVSP and MoloVol for calculating the molecular volume and surface area, as well as that of the QC software ORCA 5.0.3, were recorded separately. As shown in Table 5, CalVSP has a significantly faster computational speed than MoloVol for molecules containing fewer than 438 atoms. However, its computational efficiency markedly decreases with increasing atomic count. In contrast, the MoloVol processing speed is less dependent on the molecular size. Both CalVSP and MoloVol substantially reduce the computation time compared with that of the QC calculations. The performance degradation of CalVSP with larger systems is due primarily to increased grid point requirements and iterative computational steps associated with increased molecular size. Nevertheless, CalVSP maintains acceptable computation times above 1 min for molecular systems that contain fewer than 1,000 atoms. The calculation speed of CalVSP is much faster than that of QC calculations, making it more suitable for high-throughput calculation scenarios (such as molecular dynamics simulation trajectory analysis or high-throughput screening).

**Table 5:** Computational time analysis of molecules with different atom counts

		Time(S)		
ID	Number of Heavy Atoms	CalVSP	MoloVol	QC
Camptothecin	26	0.05	0.18	39.89
Lorlatinib	30	0.07	0.33	63.76
Pacritinib	35	0.10	0.29	68.78
Retapamulin	36	0.11	0.28	105.19
Digoxin	41	0.14	0.30	113.72
Plerixafor	36	0.13	0.50	88.58
JW48	60	0.39	0.60	159.76
Rifampin	59	0.27	0.68	222.82
Azithromycin	26	0.23	0.51	218.46
Lecithin	53	0.55	0.70	182.88
Amphotericin B	139	0.43	0.62	–
PDB:1B9E(A)	163	0.79	2.14	–
Vancomycin	101	0.53	0.98	513.7
PDB:1APH(A)	180	0.73	1.78	–
PDB:3C59(B)	209	1.07	2.50	–
PDB:1B9E(B)	245	2.24	3.37	–
PDB:1APH(B)	254	1.67	2.83	–
PDB:5OTT(B)	274	2.26	3.35	–
PDB:2M7D	152	0.93	0.89	–
PDB:1APH	438	3.02	3.32	–
PDB:5NIQ(1)	643	8.83	4.90	–
PDB:6JWE	455	6.12	2.39	–
PDB:1B9E	816	15.62	8.19	–
PDB:3C59(A)	850	17.49	8.97	–
PDB:6S8Y	894	30.46	6.03	–
PDB:1C2H	1650	119.5	9.08	–

“-” QC calculations failed. Benchmark tests were performed on Windows 11(23H2) 11th Gen Intel(R) Core(TM) i5-1155G7 CPU (8 cores @ 2.50 GHz) 16 GB (8 × 2 GB) LPDDR4X-4267 SDRAM @ 0.6 V and CalVSP compiled through Dev-C +  + Version 5.11—27 April 2015

### Validation of the PSA calculation results

**Fig. 9:**
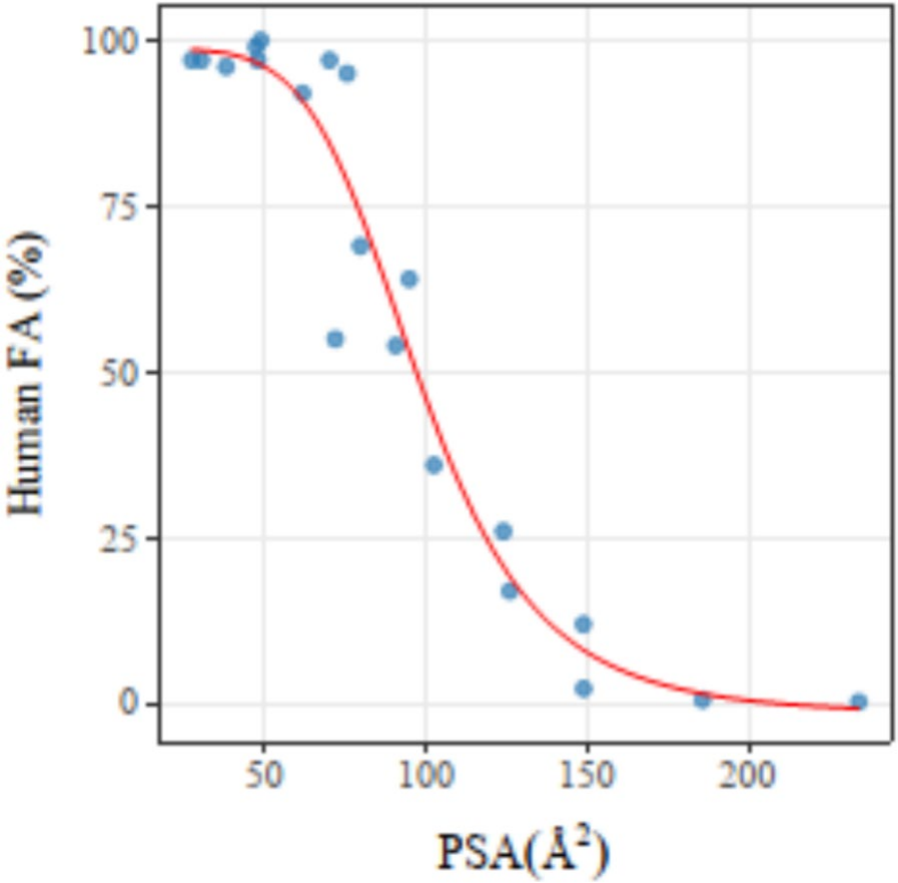
Fitting curves of the PSAs calculated via the CalVSP vs. the human FA data

To evaluate the accuracy of CalVSP in calculating the PSA, we selected reference datasets from the literature and compared the average PSA values obtained from molecular dynamics (MD) simulation trajectories with the Boltzmann-weighted average dynamic PSA (PSAd) values in Table 6 [61]. And the fit of average PSA values to human fraction absorbed data (%FA) via the Boltzmann sigmoidal curve is shown in Fig. 9 (*R*^2^ = 0.95, RMSE = 8.71%).

**Table 6:** Comparison of PSA calculated via CalVSP with literature data (PSAd)

compound	PSAd^a^/Å^2^	PSA/Å^2^	Det^b^/Å^2^
Metoprolol	53.1	48.5	4.6
Nordiazepam	45.1	47.1	− 2.0
Diazepam	33	30.2	2.8
Oxprenolol	46.8	47.7	− 0.9
Phenazone	27.1	27.1	0.0
Oxazepam	66.9	70.0	− 3.1
Alprenolol	37.1	37.8	− 0.7
Practolol	73.4	75.4	− 2.0
Pindolol	56.5	61.6	− 5.1
Ciprofloxacin	78.7	79.5	− 0.8
Metolazone	94.5	94.7	− 0.2
Tranexamic acid	69.2	71.7	− 2.5
Atenolol	90.9	90.5	0.4
Sulpiride	100.2	102.4	− 2.2
Mannitol	116.6	123.9	− 7.3
Foscarnet	115.3	125.9	− 10.6
Sulfasalazine	141.9	148.7	− 6.8
Olsalazine	141.0	148.8	− 7.8
Lactulose	177.2	185.7	− 8.5
Raffinose	242.1	234.2	7.9

^a^PSA values obtained from molecular dynamics simulation trajectories with the Boltzmann-weighted average dynamic PSA

^b^Det represents the difference between PSAd and PSA, calculated as PSAd—PSA

As shown in Table 6, there is a slight discrepancy between the calculated results and the literature data. The observed discrepancies in the calculated results arise from differences in molecular conformational sampling environments and methodologies, where the literature method employs vacuum-based conformational sampling incorporating Boltzmann distribution weighting, whereas our experimental approach applies aqueous MD simulations to derive ensemble averages directly. Nonetheless the results demonstrate a strong correlation between the PSA values derived from these trajectories and experimental drug absorption data (Fig. 9), confirming that CalVSP is a reliable tool for computing the PSAs of chemical compounds. This offers strong support for predicting the absorption and permeation properties of drug candidates[62, 63].

Molecules also change their protonated state with different environments, and the protonation state of the molecules can also change the PSA value of the molecules. Local anesthetics penetrate the cell membrane in a deprotonated form[12, 13]. We target four drug molecules with different acidic and basic properties (Nortriptyline, Pindolol, Probenecid, and Warfarin). The numerical variation of PSA value with pH value was tested.

**Fig. 10:**
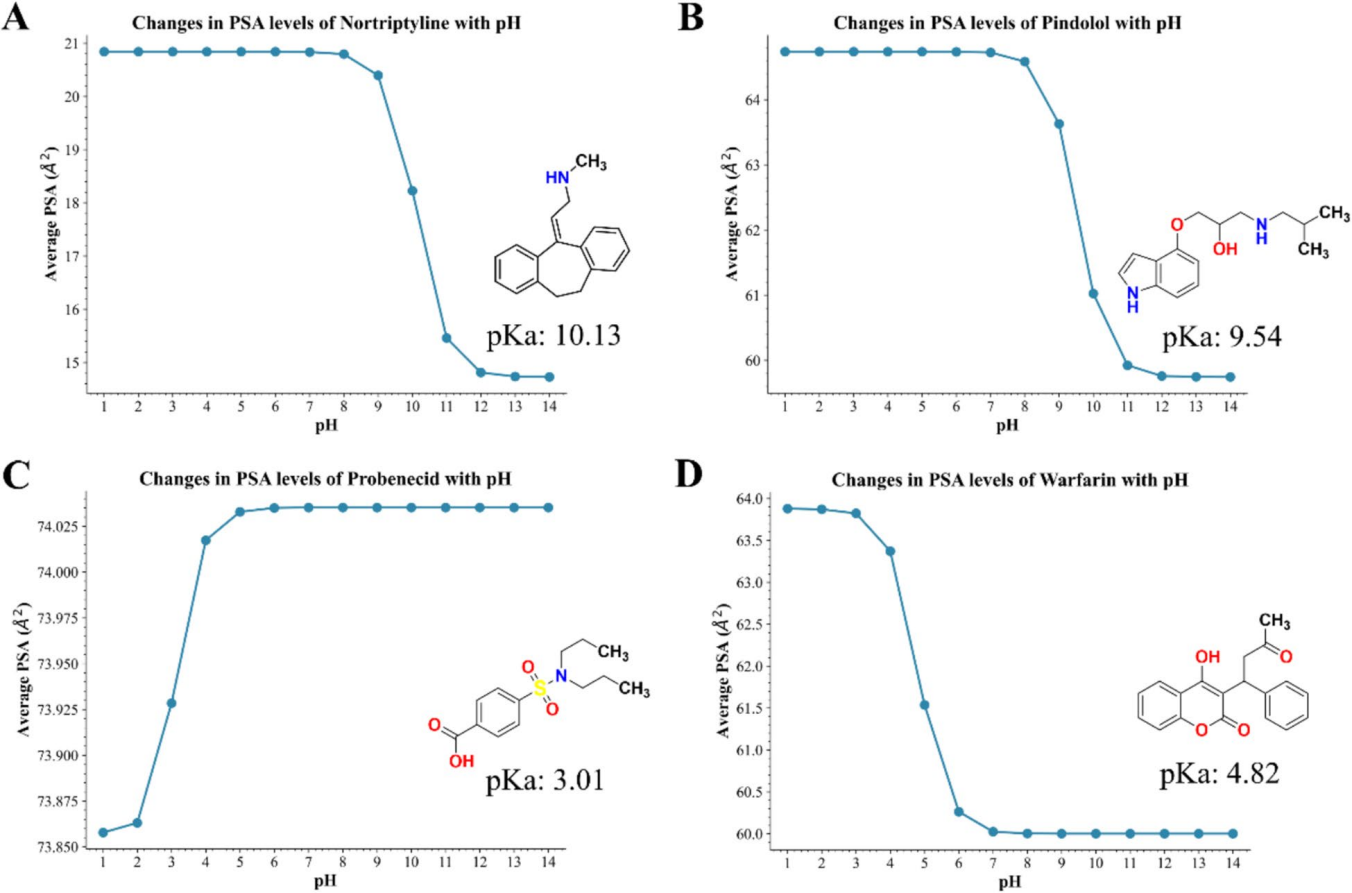
Changes in PSA of Four Drug Molecules with pH: **A** Nortriptyline; **B** Pindolol; **C** Probenecid; **D** Warfarin

From Fig. 10, it can be observed that the PSA of the four molecules undergoes significant changes with variations in pH. The magnitude of PSA change for the basic molecules Nortriptyline and Pindolol is greater than that for the acidic molecules Probenecid and Warfarin. For Probenecid, the deprotonation of its carboxylic acid group leads to an increase in PSA, which is presumably related to the greater exposure of the polar surface area of the O atoms after the COOH group loses a proton.

From the above calculations, it is evident that CalVSP can effectively reflect the changes in the PSA values of molecules during proton gain or loss. PSA is a crucial factor influencing molecular polarity, apart from atomic charge factors.

## Conclusion

Currently, numerous methods are available for calculating the molecular volume, surface area, and PSA. However, these methods may have complex calculation processes, certain requirements for computational resources, and even require multiple software supports to complete the calculation of the corresponding parameters. This paper introduces CalVSP, a tool that provides results closer to those from QC methods than commonly used tools such as PyMOL and MoloVol for molecular surface area and volume calculations. CalVSP offers a simpler workflow than do the QC methods do, achieving similar results at a lower computational cost. For complex compounds with high molecular weights that are computationally prohibitive for QC methods, CalVSP can rapidly compute the molecular volume, surface area, and PSA. Beyond standalone use, CalVSP can be integrated as a library function and combined with other methods for advanced data mining and calculations. Additionally, this study evaluated CalVSP’s accuracy in PSA calculations against PyMOL and verified its ability to accurately correlate with experimental drug absorption data. The results show that the CalVSP is a reliable and efficient tool for predicting drug properties such as absorption, distribution, metabolism, excretion, and toxicity (ADMET), offering significant support for drug discovery and development.

However, CalVSP also has certain limitations. For instance, as the number of atoms increases, the computational speed is affected. Particularly for calculations surfaces and volumes involving isosurfaces at 0.0016 a.u. and 0.002 a.u., which require smaller grid spacings, the computational speed is relatively slow and necessitates further optimization of subsequent algorithms.

## Availability and requirements

Project name: CalVSP.

Project home page: https://github.com/CalVSP/CalVSP.git

Operating system(s): Tested on Linux OS (Ubuntu 20.04 LTS) and Windows OS(11).

Programming language: C.

Other requirements: dependencies are described in the README file on the project home page.

License: MIT.

Any restrictions to use by nonacademics: None.

## Supporting information available

Test dataset. randomly selected subset of 390 3D molecular structures. Testing for High-Molecular-Weight Compounds. PSA data.

## Supplementary Information

Additional file 1.

## Data Availability

The CalVSP application is publicly available on GitHub at https://github.com/CalVSP/CalVSP.git under the MIT License. The README file in the GitHub repository provides information about how to set up and use the application. The tutorials on CalVSP are available on GitHub at https://github.com/CalVSP/CalVSP.git.
