# Supplementary material for: CalVSP: a program for analyzing the molecular surface areas, volumes, and polar surface areas
Source: J Cheminform. 2025 Dec 29;17:181. doi: 10.1186/s13321-025-01120-2 (PMC12752003; doi:10.1186/s13321-025-01120-2)
Supplement: Supplementary file 1 — Additional file 1. [file 13321_2025_1120_MOESM1_ESM.docx]

**Table S1** Grid resolution for minimizing the volume error and *V*_rec_

| *V*_rec_^a^(Å3) | Spacing of Grid Points |
| --- | --- |
| <100 | 0.40 |
| [100-360) | 0.44 |
| [360-1000) | 0.42 |
| >=1000 | 0.43 |

a Vrec, the volume of the rectangular bounding box. Spacing of Grid Points, grid spacing values minimize volume error (electron density isosurface threshold: 0.0016 a.u.).

**Table S2** Complete Computational Input Specifications for All Methods.

| Group | Functional Basis Sets |
| --- | --- |
| a | ! B97-3c noautostart miniprint nopop |
| b | ! r2SCAN-3c noautostart miniprint nopop |
| c | ! BLYP D3 def2-TZVP def2/J noautostart miniprint nopop |
| d | ! B3LYP D3 def2-TZVP(-f) def2/J RIJCOSX noautostart miniprint nopop |
| e | ! B3LYP D3 def2-TZVP def2/J RIJCOSX noautostart miniprint nopop |
| f | ! wB97M-V def2-TZVP def2/J RIJCOSX strongSCF noautostart miniprint nopop |
| g | ! PWPB95 D3 def2-TZVPP def2/J def2-TZVPP/C RIJCOSX tightSCF noautostart miniprint nopop |
| h | ! wB97X-2 D3 def2-TZVPP def2/J def2-TZVPP/C RIJCOSX tightSCF noautostart miniprint nopop |
| i | ! D4 def2-TZVPP def2/J def2-TZVPP/C RIJCOSX tightSCF noautostart miniprint nopop |
| j | ! PWPB95 D3 def2-QZVPP def2/J def2-QZVPP/C RIJCOSX tightSCF noautostart miniprint nopop |
| k | ! wB97X-2 D3 def2-QZVPP def2/J def2-QZVPP/C RIJCOSX tightSCF noautostart miniprint nopop |
| l | ! D4 def2-QZVPP def2/J def2-QZVPP/C RIJCOSX tightSCF noautostart miniprint nopop |
| m | ! DLPNO-CCSD(T) normalPNO RIJK cc-pVTZ cc-pVTZ/JK cc-pVTZ/C tightSCF noautostart miniprint nopop |
| n | ! DLPNO-CCSD(T) tightPNO RIJK cc-pVTZ cc-pVTZ/JK cc-pVTZ/C tightSCF noautostart miniprint nopop |
| o | ! CCSD(T) cc-pVTZ tightSCF noautostart miniprint nopop |
| p | B3LYP/6-31G |
| q | B3LYP/6-31G** |
| r | B3LYP/6-311G |
| s | B3LYP/6-311G** |


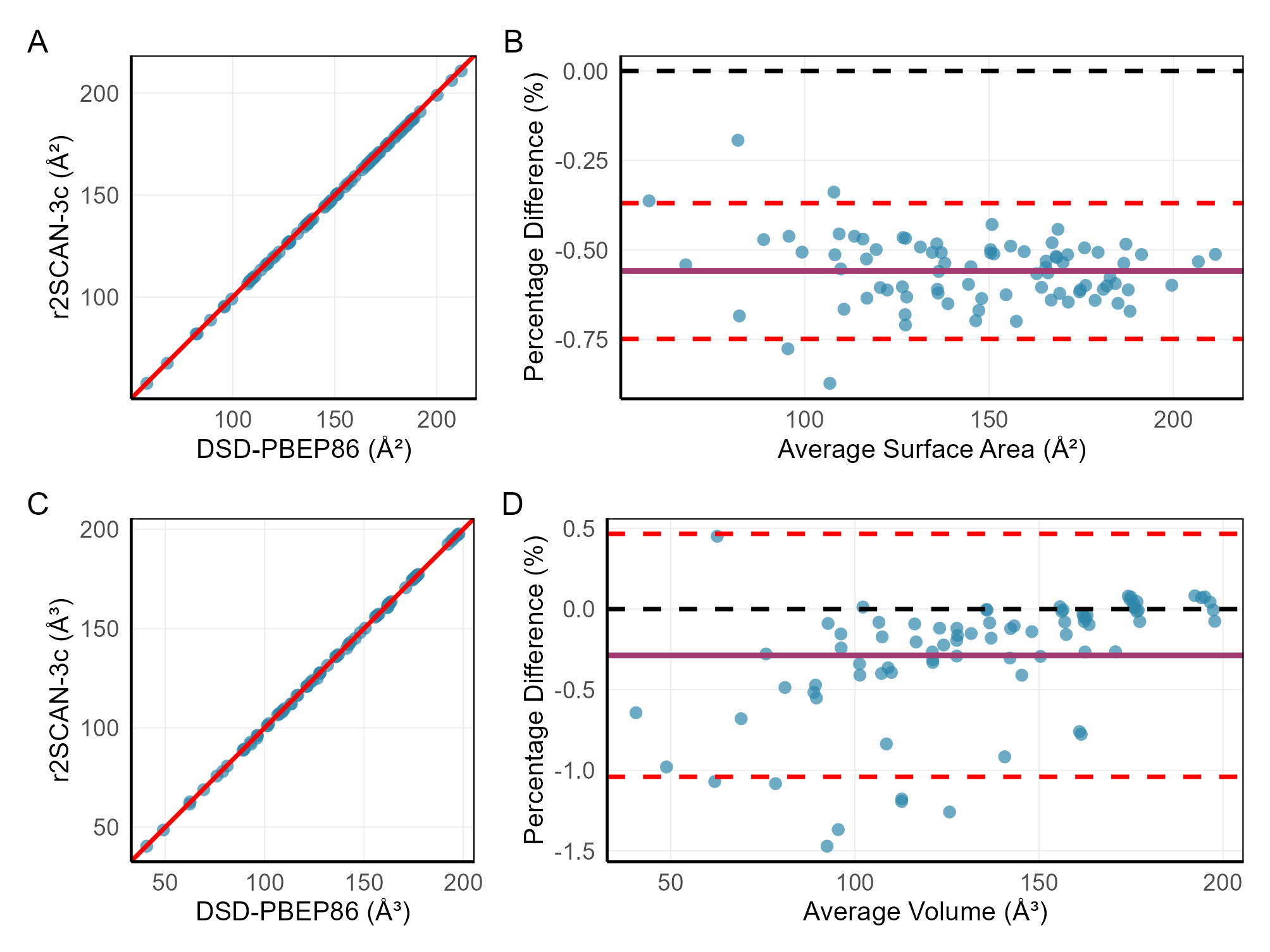


**Fig.S1** Comparison of molecular volumes and surface areas calculated using the DSD-PBEP86 functional and the r2SCAN-3c functional (n=82; electron density isosurface threshold: 0.0016 a.u.). (A) correlation scatter plot between DSD-PBEP86 and r2SCAN-3c of vdW surface areas (*R^2^*=0.999); (B) Bland-Altman analysis of surface area agreement (pink solid line: mean difference -0.56%; red dashed lines: 95% limits of agreement -0.75% to -0.37%). (C) correlation scatter plot between DSD-PBEP86 and r2SCAN-3c of vdW volumes (*R^2^*=0.999); (D) Bland-Altman analysis of volume agreement showing a mean difference of -0.29% (95% limits of agreement: -1.04% to 0.47%).

To quantify the changes, we first calculated the volumes (Va) and surface areas (Sa) of individual C, N, O, and Cl atoms using high-level quantum chemical methods (ORCA, CCSD(T)/cc-pVTZ). And the calculation parameters of small molecules are consistent with those of atoms. The calculated atomic volume and surface area parameters are shown in Table S3. The corresponding molecular volume and surface area data are shown in Table S4.

**Table S3** Calculated Atom Volumes and Surface Areas

| Atoms | Volume(Å^3^) | Surface(Å²) |
| --- | --- | --- |
| C | 28.708 | 45.565 |
| N | 23.500 | 39.708 |
| O | 20.352 | 36.175 |
| Cl | 36.389 | 53.231 |

Atom volumes and surface areas calculated by Multiwfn 3.8 and electron density isosurface threshold of 0.001 a.u..

**Table S4** Comparison of QC and Atomic Sum Methods for Molecular Volumes and Surface Areas

| Mol | V(Å^3^) | S(Å²) | Vcount(Å^3^) | Scount(Å²) | detV(Å^3^) | detS(Å²) |
| --- | --- | --- | --- | --- | --- | --- |
| N_2_ | 35.476 | 52.852 | 47.0 | 79.416 | 24.5% | 33.4% |
| CO | 36.648 | 54.159 | 49.06 | 81.740 | 25.3% | 33.7% |
| CO_2_ | 44.583 | 63.821 | 69.412 | 117.915 | 35.8% | 45.9% |
| O_2_ | 32.073 | 49.831 | 40.704 | 72.350 | 21.2% | 31.1% |
| CCl_4_ | 126.959 | 134.498 | 174.264 | 258.489 | 27.1% | 48.0% |
| COCl_2_ | 87.159 | 103.666 | 121.838 | 188.202 | 28.5% | 44.9% |

QC, Quantum Chemistry; Volume(V) and Surface(S), Obtained through QC calculations (Multiwfn 3.8, electron density isosurface threshold of 0.001 a.u..); Vcountc and Scountc calculated as equation, $V_{count}=\sum_{i=1}^{n} (V_{a})_{i}$,$S_{count}=\sum_{i=1}^{n} (S_{a})_{i}$; DetV and DetS are calculated according to the following equation, $\det V=\frac{V_{count}-V}{V_{count}}*100\%$, $\det S=\frac{S_{count}-S}{S_{count}}*100\%$.

**Table S5** Comparison between CalVSP and QC Calculation Methods in Calculating Molecular Surface Area and Volume (0.002 a.u.)

| Metric | Surface | Volume |
| --- | --- | --- |
| MSE | 20.6±0.3 (Å^2^)^2^ | 13.6±0.3 (Å^3^)^2^ |
| RMSE | 4.54±0.04 Å^2^ | 3.69±0.04 Å^3^ |
| MAE | 3.58±0.03 Å^2^ | 2.88±0.02 Å^3^ |
| MAPE | 1.027±0.008 % | 0.837±0.007 % |
| Pearson’s r | 0.99873±0.00003 | 0.99927±0.00002 |
| *R*^2^ | 0.99722±0.00006 | 0.9985±0.00003 |

0.002, electron density isosurface threshold of 0.002 a.u..


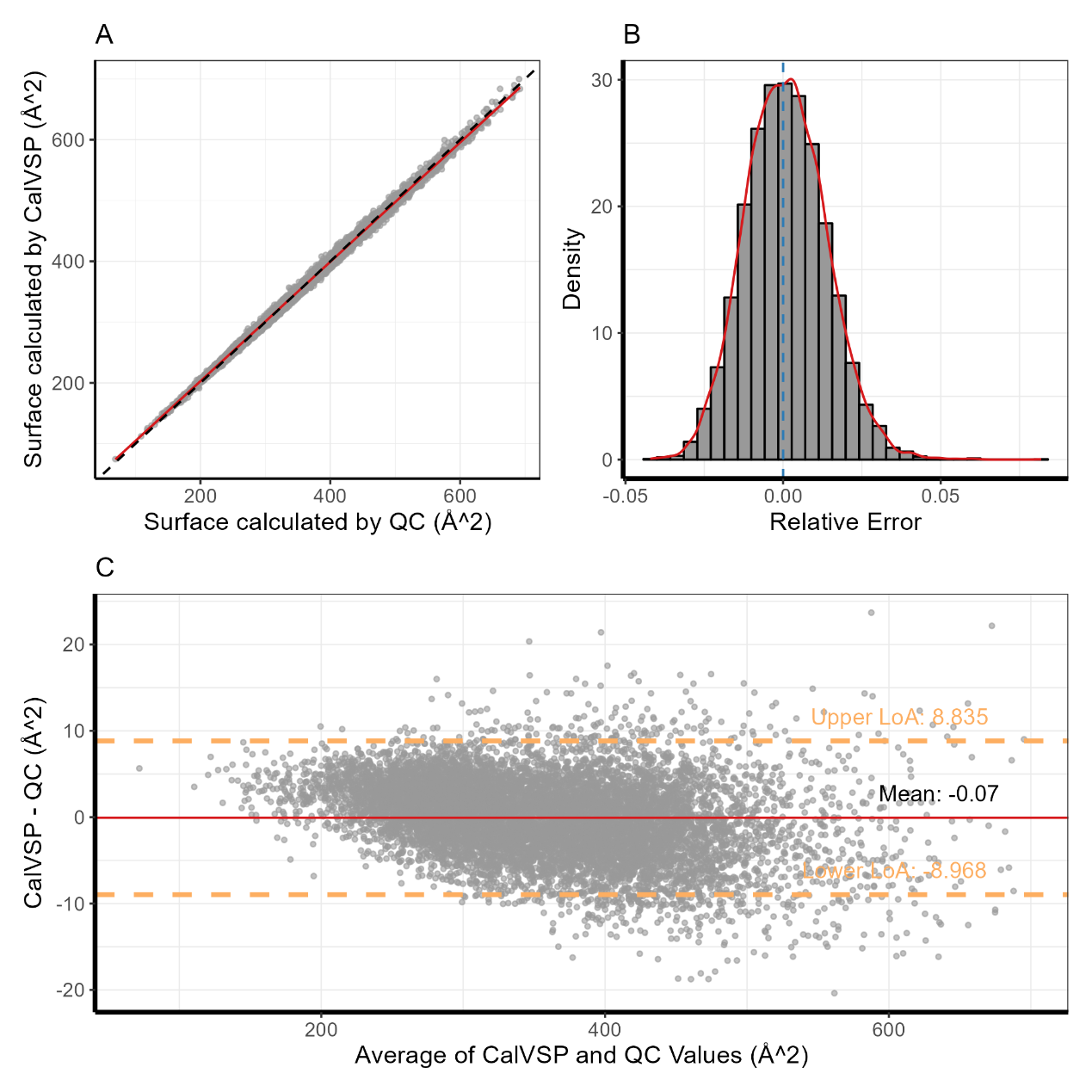


**Fig. S****2** Computational validation of van der Waals surface metrics (electron density isosurface threshold of 0.002 a.u.): (A) QC-calculated vdW surfaces frequency distribution; (B) correlation scatter plot between QC-derived and CalVSP-calculated vdW surfaces; (C) relative error frequency distribution of surface area computations; (D) Bland‒Altman analysis of methodological agreement;


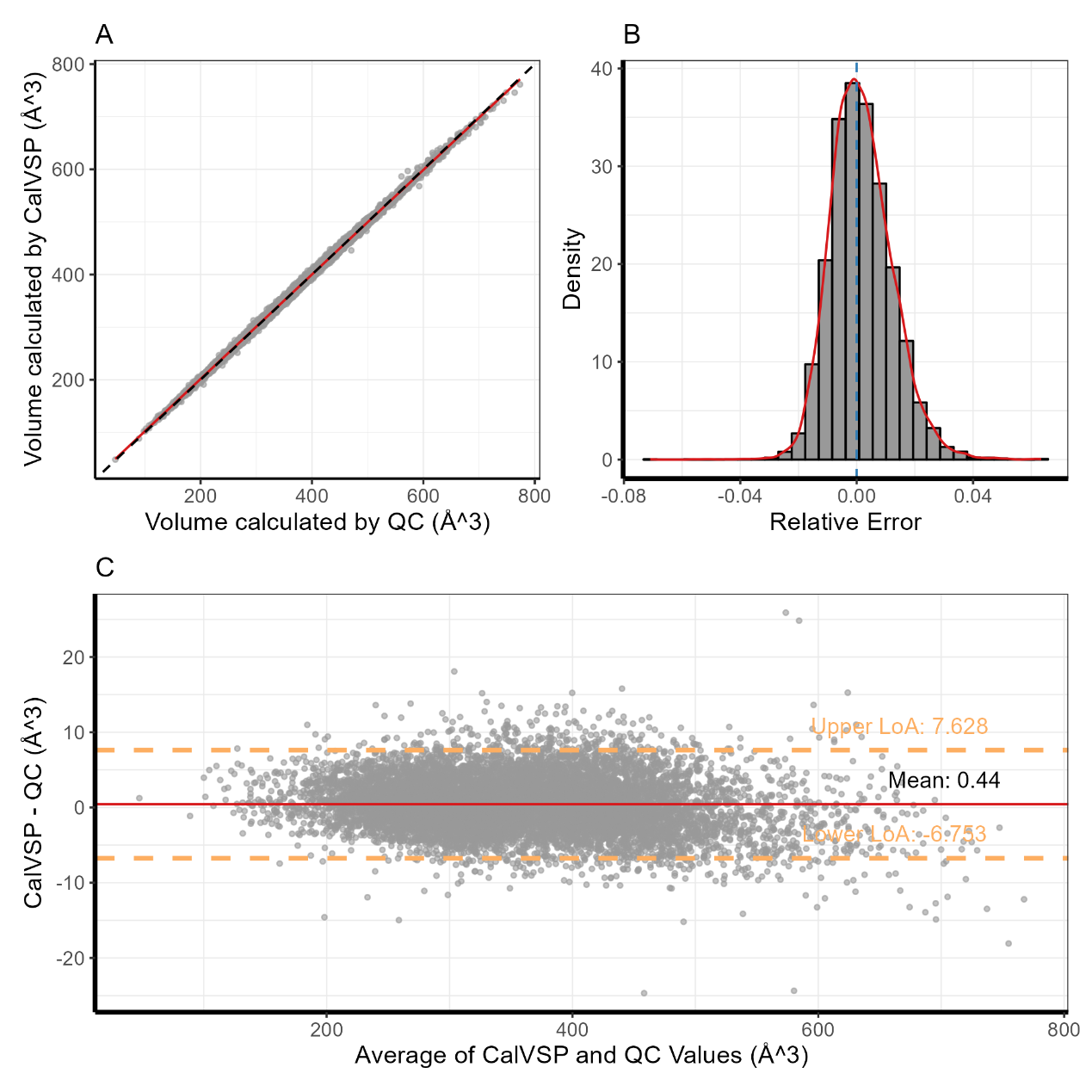


**Fig. S3** Computational validation of van der Waals surface metrics (electron density isosurface threshold of 0.002 a.u.): (A) QC-calculated vdW volumes frequency distribution ;  **(**B) Scatter plot comparing molecular van der Waals volumes derived from QC calculations and CalVSP; (C) Relative error distribution of CalVSP-calculated van der Waals volumes relative to that of QC; (D) Bland‒Altman plot assessing the agreement between the van der Waals volumes calculated via QC and CalVSP;

**Table S6** Comparison between CalVSP and QC Calculation Methods in Calculating Molecular Surface Area and Volume (0.0016 a.u.)

| Metric | Surface | Volume |
| --- | --- | --- |
| MSE | 23.7±0.4 (Å^2^)^2^ | 17.6±0.3 (Å^3^)^2^ |
| RMSE | 4.87±0.04 Å^2^ | 4.19±0.04 Å^3^ |
| MAE | 3.81±0.03 Å^2^ | 3.29±0.03 Å^3^ |
| MAPE | 1.061±0.008 % | 0.895±0.007 % |
| Pearson’s r | 0.99855±0.00003 | 0.99914±0.00002 |
| *R*^2^ | 0.99686±0.00006 | 0.99827±0.00004 |


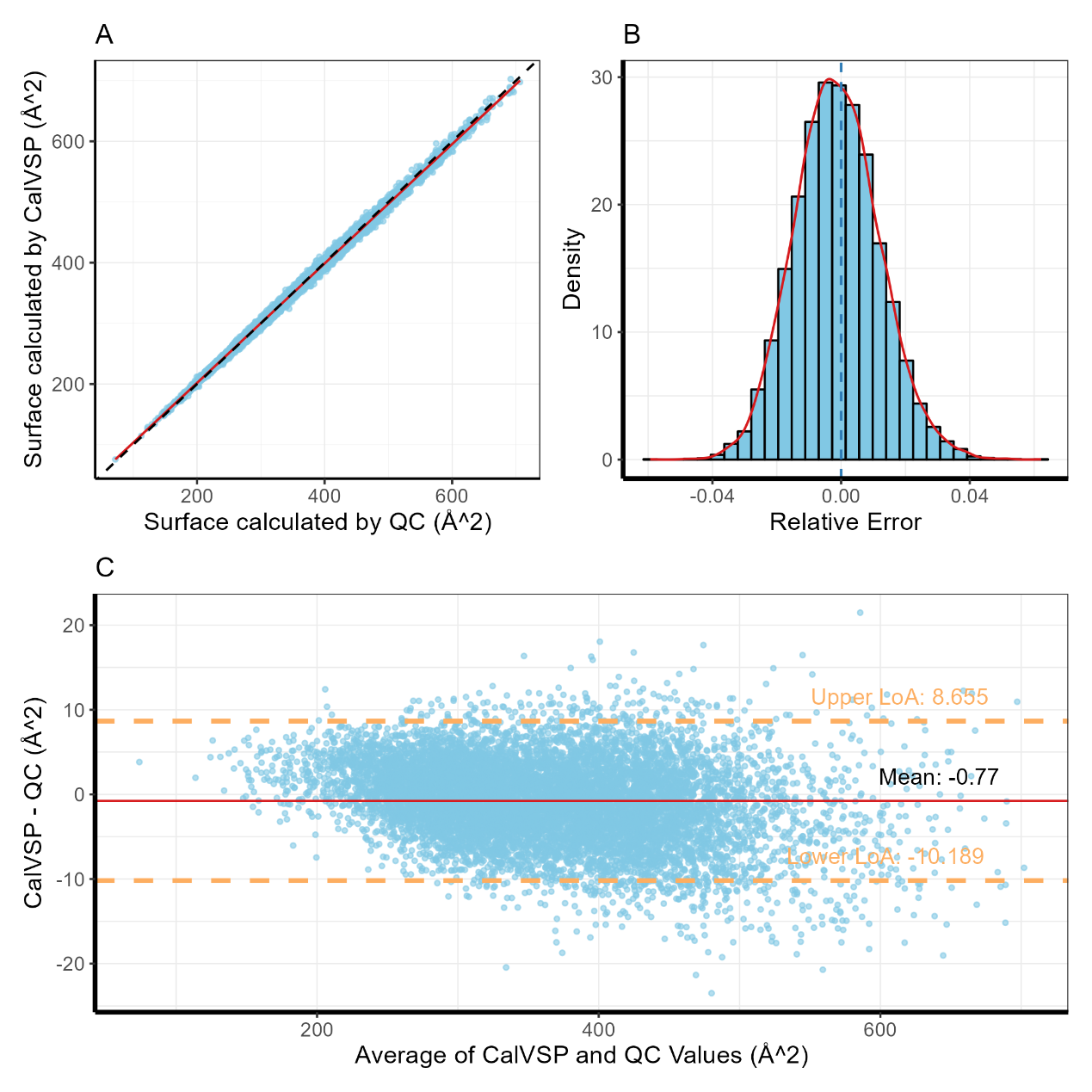


**Fig. S4** Computational validation of van der Waals surface metrics (electron density isosurface threshold of 0.0016 a.u.): (A) QC-calculated vdW surfaces frequency distribution; (B) correlation scatter plot between QC-derived and CalVSP-calculated vdW surfaces; (C) relative error frequency distribution of surface area computations; (D) Bland‒Altman analysis of methodological agreement;


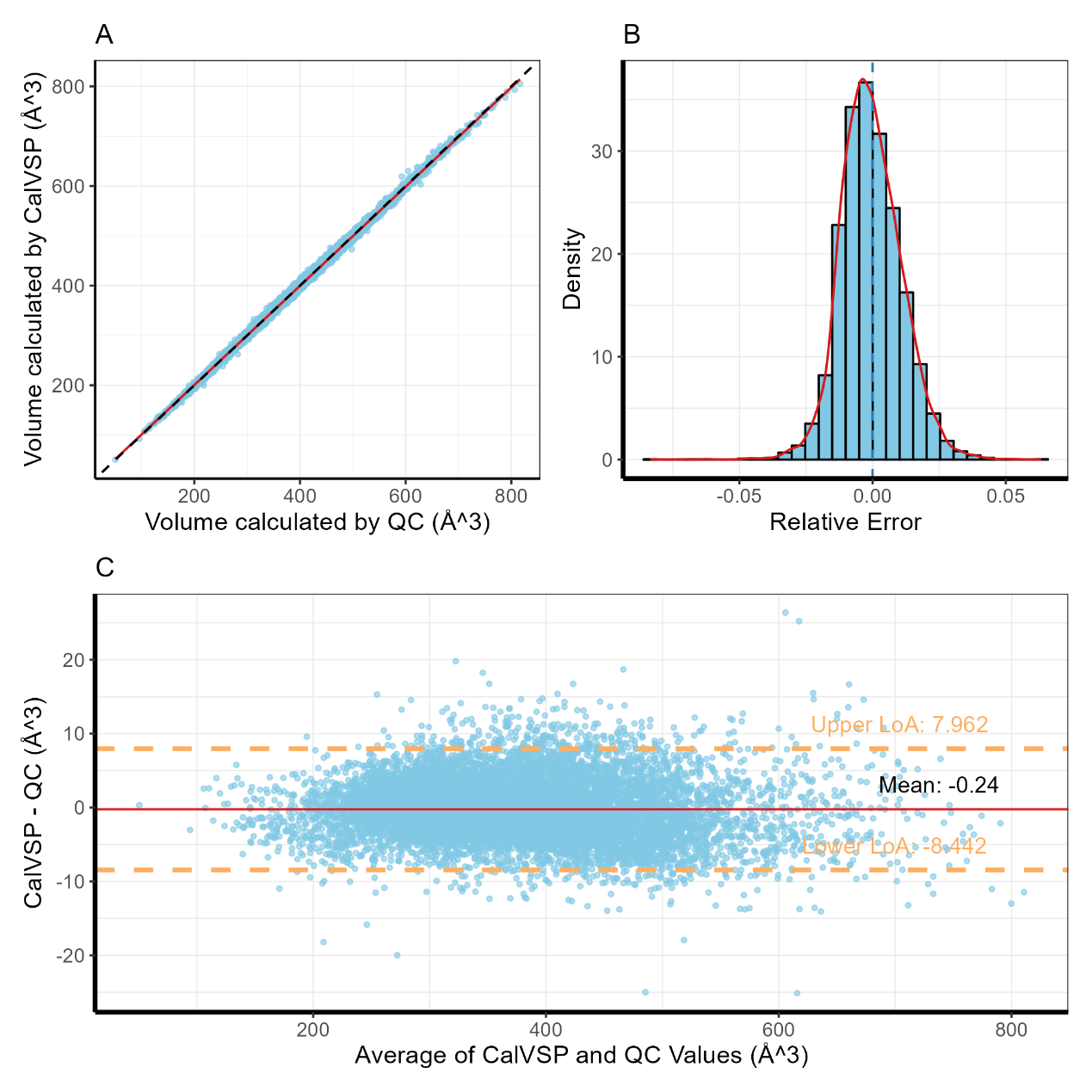


**Fig. S5** Computational validation of van der Waals surface metrics (electron density isosurface threshold of 0.0016 a.u.): (A) QC-calculated vdW volumes frequency distribution ;  **(**B) Scatter plot comparing molecular van der Waals volumes derived from QC calculations and CalVSP; (C) Relative error distribution of CalVSP-calculated van der Waals volumes relative to that of QC; (D) Bland‒Altman plot assessing the agreement between the van der Waals volumes calculated via QC and CalVSP;
